# Enhanced Stability of Sodium‐Ion Batteries by Controlling the Synthesis Process of Binary Metal Sulfides

**DOI:** 10.1002/smll.202412776

**Published:** 2025-03-27

**Authors:** Wenbo Qiu, Zidong Wang, Huaping Zhao, Yonglong Sheng, Guosheng Shao, Yong Lei

**Affiliations:** ^1^ Institute of Nanochemistry and Nanobiology School of Environmental and Chemical Engineering Shanghai University Shanghai 200444 China; ^2^ Fachgebiet Angewandte Nanophysik Institut für Physik & IMN MacroNano Technische Universität Ilmenau 98693 Ilmenau Germany; ^3^ School of Materials Science and Engineering Zhengzhou University Zhengzhou 450001 China

**Keywords:** anode, binary metal sulfides, enhanced stability, sodium‐ion battery, synthesis process

## Abstract

Binary metal sulfides hold significant promise as anode materials for advanced sodium‐ion batteries (SIBs), but their application is often limited by rapid capacity degradation and slow reaction kinetics. While carbon composites are frequently used to address these issues, the influence of the sequence of carbonization and sulfidation on anode performance has been largely overlooked. To bridge this gap, Co‐Sn sulfides are synthesized through various processes to examine the impact of synthesis methods on material properties. Among these, the one‐step synthesized CSS‐C1 exhibits enhanced sodium‐ion kinetics and excellent stability. It delivers a capacity of 220.4 mAh g^−1^ at an ultra‐high current density of 20 A g^−1^ and maintained 389 mAh g^−1^ over 2300 cycles at 10 A g^−1^. When assembled into full‐cell devices (CSS‐C1||Na_3_V_2_(PO_4_)_3_), it demonstrates stable capacity retention for over 900 cycles, establishing it as a highly stable and efficient anode material for SIBs.

## Graphical abstract

For sodium‐ion battery sulfide anodes, carbon composites are commonly used to address stability and rate performance issues. However, the impact of the carbonization‐sulfidation sequence on anode performance remains underexplored. This study reveals that while carbon composites enhance stability and rate capability, optimizing the synthesis sequence can further maximize these improvements.

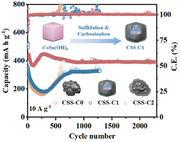

## 1 Introduction

Although lithium‐ion batteries (LIBs) are widely used as a common energy storage technology in daily life,^[^ ^1^, ^2^, ^3^ ^]^ there is an urgent need for new alternatives. Sodium‐ion batteries (SIBs) present a promising option for the post‐lithium era. Sodium is abundant in the crust, which regarded as a potential complementary technology to lithium‐ion batteries due to their wide availability of raw materials and cost advantages, especially in the short term to help mitigate the impact of uneven distribution of lithium resources and market price fluctuations on the battery industry chain. Additionally, sodium‐ion batteries have shown good feasibility in specific application scenarios (e.g., large‐scale energy storage systems), which can reduce the dependence on lithium resources to a certain extent.^[^ ^4^, ^5^ ^]^ There are a lot of similar properties between sodium and lithium, including comparable redox potentials. Benefiting from these similarities, SIBs, and LIBs share a largely identical structure and storage mechanism. Despite these advantages, SIBs have not yet achieved the same level of commercial success as LIBs, largely due to fundamental differences in ionic carriers. The larger ionic radius of sodium causes structural collapse in electrode materials, negatively impacting cycling stability and capacity retention. In addition, many electrode materials commonly used in LIBs, such as graphite and non‐laminated metal oxides, are unsuitable for SIB applications.^[^ ^6^ ^]^ The overcome these challenges, discovering high‐performance anode materials is critical for advancing SIB technology in the post‐lithium era.^[^ ^7^, ^8^, ^9^ ^]^

Transition metal sulfides (TMS) based on multi‐electron transfer storage mechanisms have been demonstrated to exhibit significantly higher specific capacity than inserted carbon‐based materials, making them promising candidates for advancing SIB anode materials.^[^ ^10^, ^11^, ^12^, ^13^, ^14^, ^15^ ^]^ Cobalt and tin sulfides are notable examples. However, they often suffer from short cycle life and rapid capacity decay due to the structural collapse during charging and discharging. To address these challenges, various improvement strategies have been developed. One effective approach is interface engineering. The composite of two elements produces rich phase interfaces and small crystalline regions compared to a simple single‐element, resulting in improved electrical conductivity and specific capacity.^[^ ^16^, ^17^, ^18^, ^19^ ^]^ Another widely adopted method is carbon coating, which could suppress the issue of polysulphides during battery reactions. The polysulfide shuttle effect is a major factor leading to capacity loss and stability degradation. Carbon‐based materials can effectively prevent the leakage of polysulfides while also mitigating material volume expansion and improving electron transport efficiency.^[^ ^20^, ^21^, ^22^, ^23^ ^]^ Moreover, the carbon coating helps to alleviate volume changes during the reaction, enhancing stability, which is particularly crucial for tin‐based materials. Hence, carbon‐coated two‐element (Co, Sn) sulfides emerge as promising candidates for advanced SIB anode materials. However, their preparation including sulfurization and carbonization of the precursors, where the sequence of these processes should be important. Different processing sequences may result in poor contact between the active material and the carbon coating, thereby hindering electron transport and exhibiting unsatisfactory performance enhancement.^[^ ^24^, ^25^ ^]^ Moreover, the inconsistent synthesis sequences reported in the publications imply the inadequate investigation of relationship between the synthesis sequences and performance improvements in these materials. To the best of our knowledge, the impact of processing sequence on anode material performance has not yet been systematically reported. Therefore, it is of significant interest to explore and control the synthesis process sequences for binary metal sulfides to optimize their properties and performance.

Based on these concepts, we prepared carbon coated cobalt‐tin sulfides by using different synthesis processes to explore how the synthesis process influences SIB anode performance. By employing a series of characterization techniques and electrochemical investigation, we have found that rational optimization of the synthesis steps can maximize the stability improvement of carbon coating. The products treated by combining sulfidation and carbonization offer better performance due to faster kinetic processes and electron transfer. The prepared SIB anode exhibited a reversible capacity of 575 mAh g^−1^ at 0.5 A g^−1^. Even after 2300 cycles at a current density of 10 A g^−1^, it retains 90.2% of its capacity, demonstrating a high stability. When assembled into a full cell, it can be stabilized at 130.5 mAh g^−1^ after 900 cycles.

## 2 Results and Discussion

### 2.1 Synthesis and Characterizations of Materials

**Figure 1:**
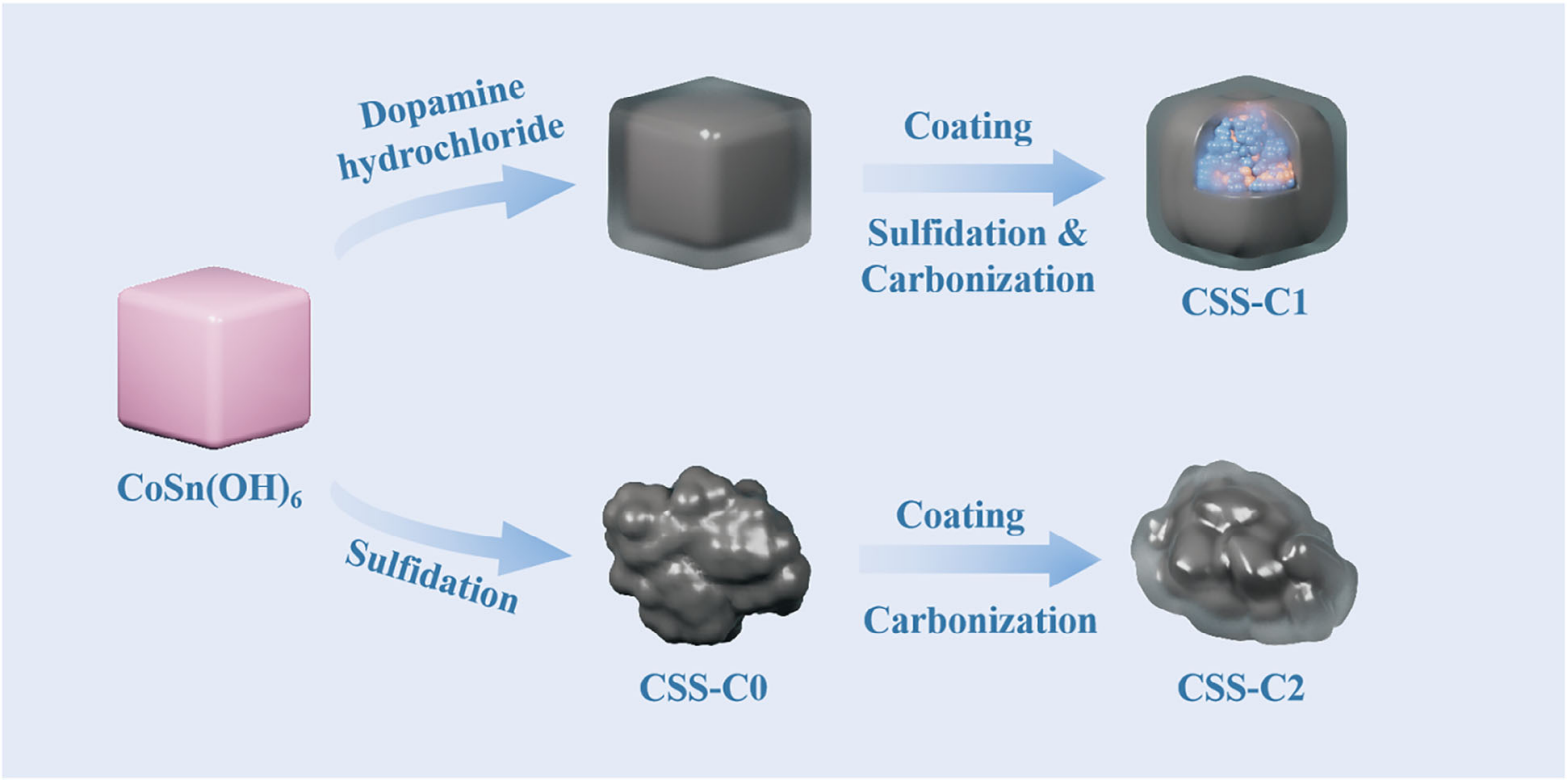
Schematic diagram of the synthesis process of CSS‐C0, CSS‐C1, and CSS‐C2, respectively.

To explore the effect of the order of sulfidation and carbonization, the samples were prepared by a simple two‐step process. **Figure** **1** shows a schematic diagram of the synthesis process. The same precursors CoSn(OH)_6_ nanocubes were obtained by a co‐precipitation method and different final products were obtained by different following treatments. Product with a sulfidation treatment only is called CSS‐C0. Product treated by a combined sulfidation and carbonization is named as CSS‐C1. And CSS‐C2 is the product of separate sulfidation and carbonization.

**Figure 2:**
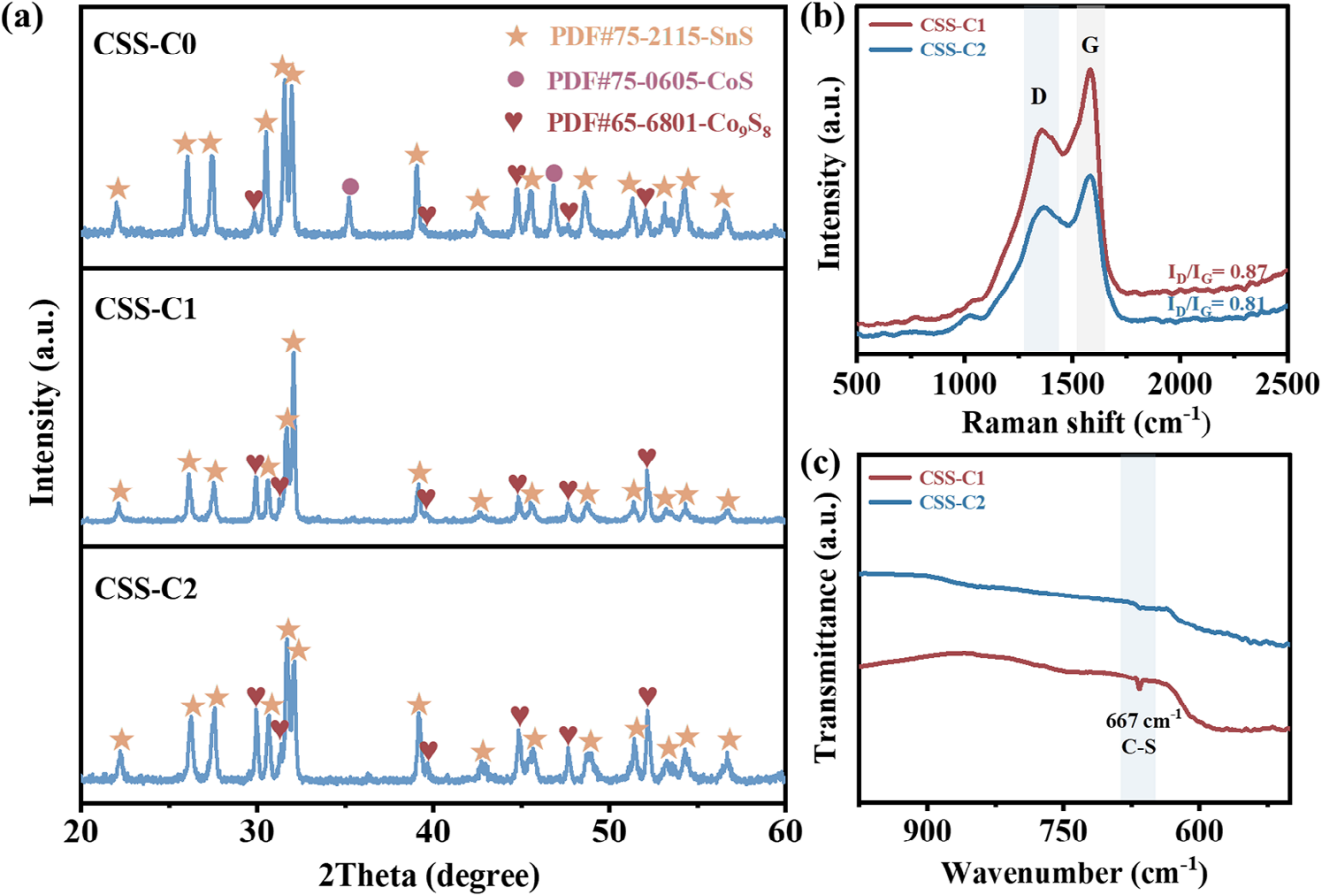
a) XRD patterns of CSS‐C0, CSS‐C1, and CSS‐C2, respectively. b) Raman spectra and c) FTIR spectra of CSS‐C1 and CSS‐C2, respectively.

The crystal structures of final products have been further analyzed by X‐ray diffraction (**Figure** **2**a). While all three XRD patterns are similar, slight differences can be still observed. All samples correspond to SnS (PDF#75‐2115) and Co_9_S_8_ (PDF#65‐6801), but CSS‐C0 additionally contains two cobalt sulfide phases: Co_9_S_8_ and CoS (PDF#75‐0605). The absence of CoS in the other samples is likely due to chemical interactions between carbon and sulfur during the carbonization process, which are accompanied by sulfur evaporation.^[^ ^26^, ^27^ ^]^ The different synthesis processes also result in the (400) plane of SnS in CSS‐C1 exhibiting improved crystallinity, which may enhance its capacity and stability for sodium‐ion storage.^[^ ^28^, ^29^ ^]^ Raman analysis (Figure 2b) reveals a peak at ≈1355 cm⁻¹ (D band), representing amorphous carbon, and another peak at ≈1586 cm⁻¹ (G band), associated with ordered graphitic carbon. Their intensity ratio (I_D_/I_G_) is 0.87 for CSS‐C1 and 0.81 for CSS‐C2, indicating that the one‐step synthesis process reduces structural defects and increases graphitization degree, thereby enhancing conductivity.^[^ ^18^, ^26^, ^30^ ^]^ The carbon content was determined by TGA (Figure S1, Supporting Information). The decomposition involves a multi‐step reaction and the weight increase can be attributed to the oxidation of Co_9_S_8_ and SnS. The carbon content of CSS‐C1 is 7%, which is 10% of CSS‐C2.^[^ ^31^, ^32^, ^33^ ^]^

FTIR analysis (Figure 2c) shows a C─S bond in CSS‐C1 absent in CSS‐C2, indicating strong interaction between sulfur and carbon in CSS‐C1.^[^ ^34^, ^35^, ^36^ ^]^ C─S bonding facilitates electron transfer, which can improve rate performance and cycling stability.

**Figure 3:**
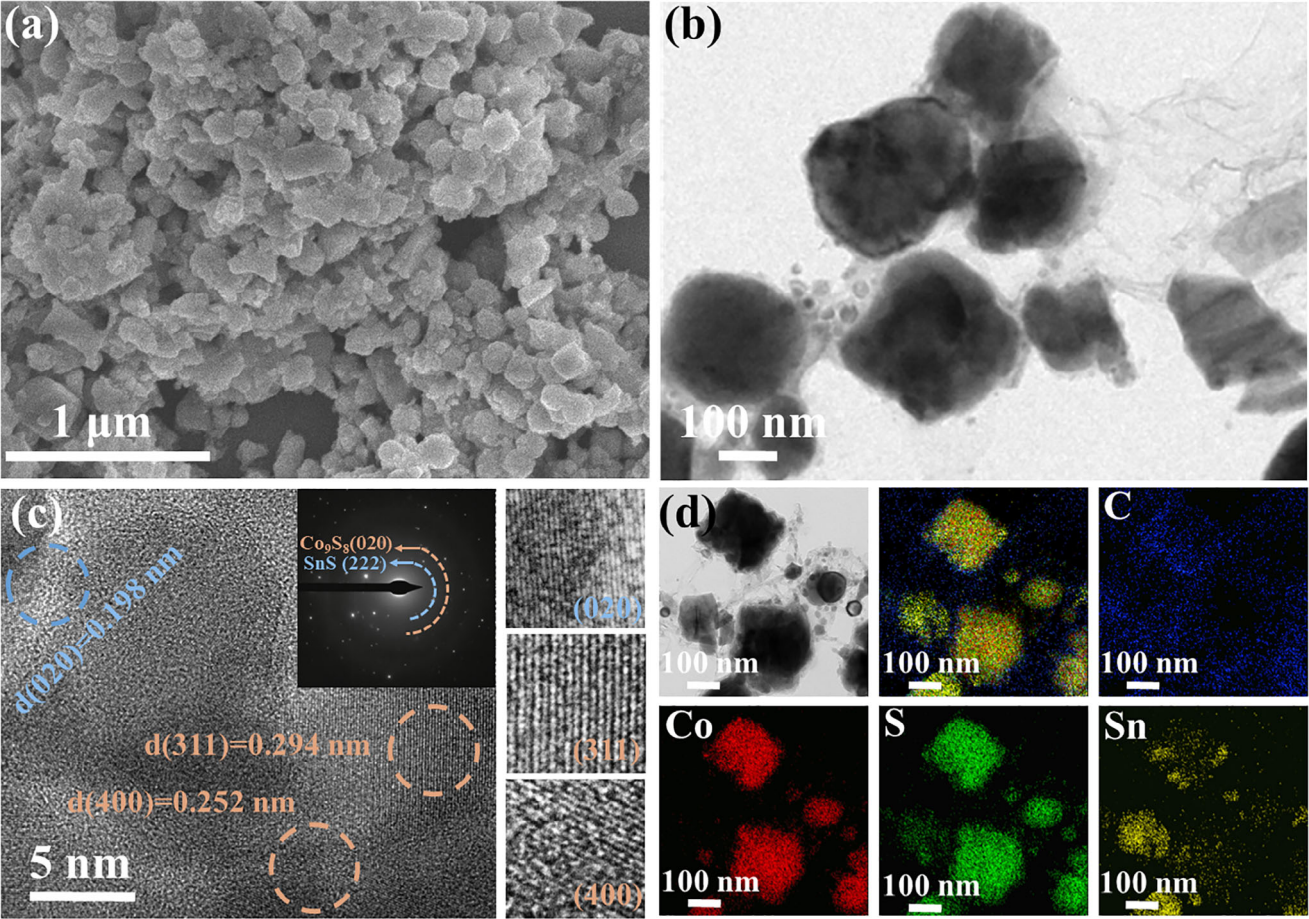
a) SEM image, b) TEM image, c) HRTEM image with corresponding lattice spacing and SAED pattern, and d) element mapping images of CSS‐C1, respectively.

Figure S1 (Supporting Information) reveals that the precursor exhibits a characteristic cubic morphology. After different treatments, CSS‐C1 demonstrates better retention of its original morphology (**Figure** **3**a), due to the constraining effect of the carbon precursors. In contrast, the morphological disruption observed in CSS‐C0 and CSS‐C2 (Figure S2, Supporting Information) is attributed to the unregulated reactions of the two metal elements during the sulfidation process without carbon precursors. Without a coating to regulate growth, nucleation on the precursor surface leads to unrestricted growth in all directions, resulting in altered morphology.^[^ ^37^ ^]^ Additionally, this one‐step synthesis process successfully retains the original morphology. As suggested by previous work, this retention is likely to significantly enhance the stability and efficiency of sodium‐ion storage.^[^ ^12^ ^]^ The preservation of the morphology can be further supported by the TEM image (Figure 3b), which clearly shows the encapsulation of the outer layer. The lattice spacings (Figure 3c) of 0.198, 0.294, and 0.252 nm, correspond to the (020) plane of SnS and the (311) and (400) plane of Co_9_S_8_, respectively. The selected area electron diffraction (SAED) pattern of CSS‐C1 also shows the coexistence of SnS and Co_9_S_8_. This result can be corresponded to the previous XRD analysis. Furthermore, the distribution of elements shown in Figure 3d is uneven, which may be due to different growth rates of different elements during heating. The distribution of carbon elements reveals that carbon is mainly coated as the outer layers, creating an encapsulating structure. The carbon coating effectively mitigates volume changes during sodium ion storage, improving the stability of active materials.

**Figure 4:**
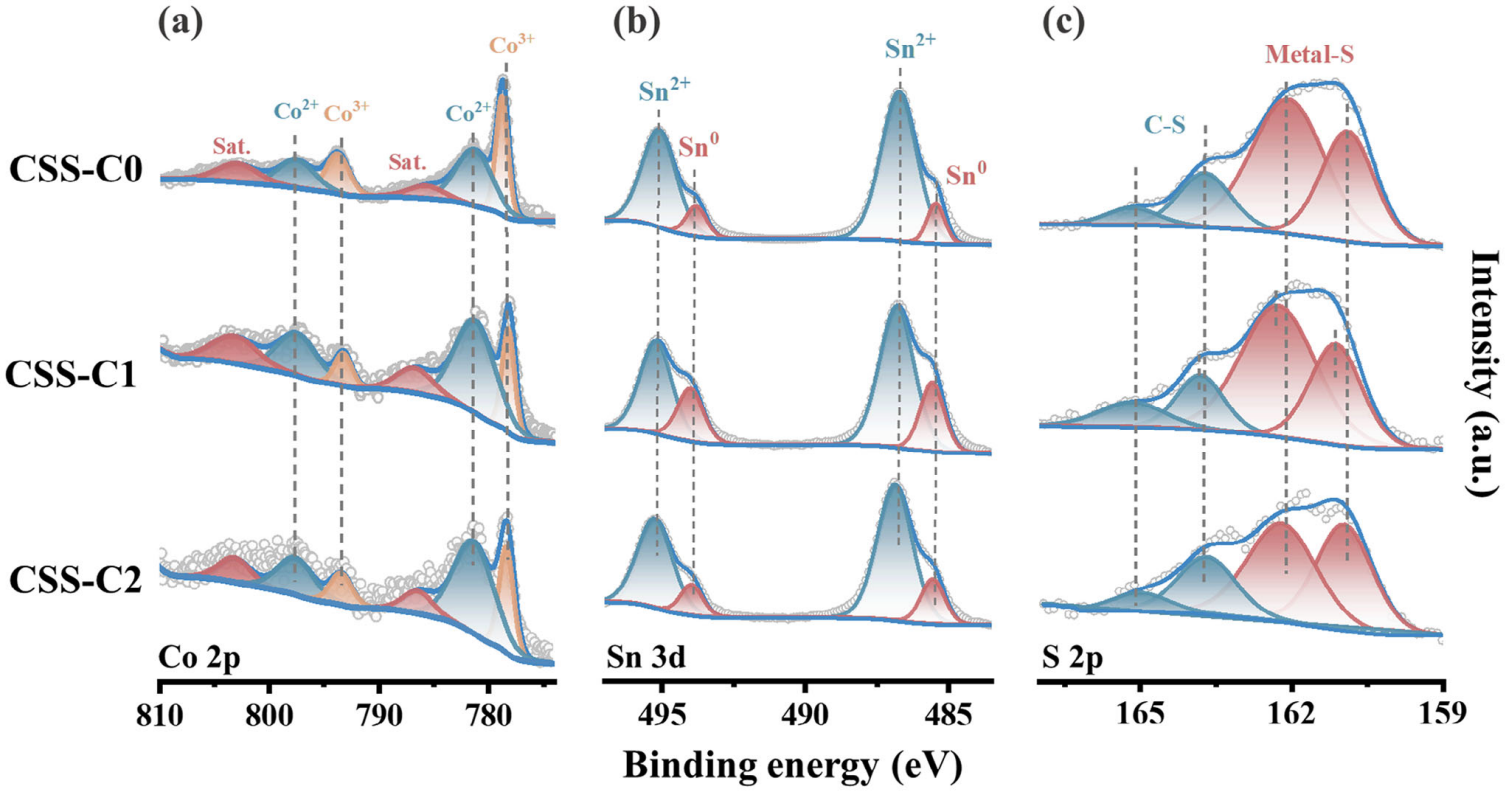
High‐ resolution XPS spectrum of a) Co 2p, b) Sn 3d, and c) S 2p for CSS‐C0, CSS‐C1, and CSS‐C2, respectively.

XPS analysis was employed to investigate the elemental compositions and surface chemical states of CSS‐C0, CSS‐C1, and CSS‐C2, respectively. Figure S3 (Supporting Information) confirms the presence of Co, Sn, S, C, and O, while the detected O can be contributed to surface oxidation.^[^ ^38^ ^]^ The fine spectra of different elements are shown in **Figure** **4**. In the Co 2p spectrum the peaks at 778.3 eV (Co^3+^) and 781.6 eV (Co^2+^) are attributed to Co 2p_3/2_, while those at 797.7 eV (Co^3+^) and 797.5 eV (Co^2+^) correspond to Co 2p_1/2_.^[^ ^39^, ^40^ ^]^ The characteristic satellite peak at 786.1 eV also confirms the existence of Co^2+^. The one‐step strategy markedly boosts the relative intensity of Co^2+^ in the material, indicating a higher Co^2+^ ratio, which facilitates enhanced reactions.^[^ ^41^ ^]^ As shown in Sn 3d spectrum, the existence of Sn^0^ are confirmed by peaks at 485.5 eV (Sn 3d_5/2_) and 493.8 eV (Sn 3d_3/2_), while peaks at 486.7 eV (Sn 3d_5/2_) and 495.1 eV (Sn 3d_3/2_) verify the existence of Sn^2+^.^[^ ^42^, ^43^, ^44^ ^]^ The increased Sn⁰ intensity in CSS‐C1 can be attributed to the influence of carbon‐introduced thermal reduction. Diverse valence states mean more active reactions, favoring sodium ion storage. The S 2p spectrum reveals peaks at 161.1 and 162.3 eV, which are associated with Metal‐S bonds, while the peaks at 163.5 and 164.8 eV indicate the presence of C‐S bonds.^[^ ^43^, ^45^, ^46^ ^]^ The S 2p peaks shift to high binding energies, indicating an interaction occurred in CSS‐C1 and CSS‐C2, facilitating enhanced electron transfer.^[^ ^22^ ^]^ Additionally, the peak near 163 eV for CSS‐C1 shifts toward higher binding energy compared to CSS‐C2, indicating a stronger C─S interaction in CSS‐C1. The main peak of C 1s (Figure S3, Supporting Information) can be observed as C─C/C═C, C─O/C─S, and C═O.^[^ ^47^ ^]^

### 2.2 Electrochemical Performance Characterizations

**Figure 5:**
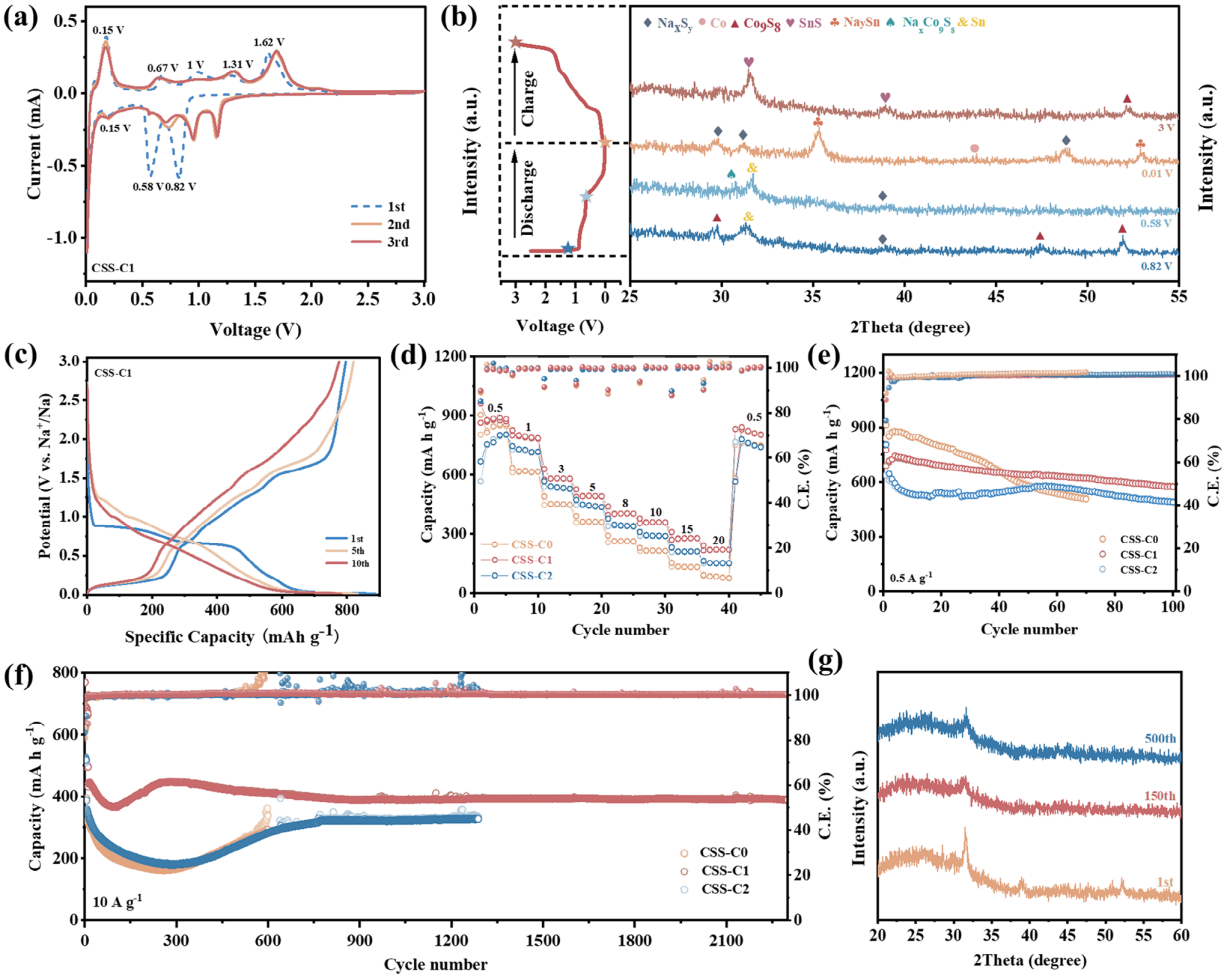
a) CV curves of CSS‐C1 at 0.1 mV s^−1^ within voltage ranges of 0.01–3 V. b) Ex situ XRD patterns corresponding to different voltages. c) Charge/discharge profiles of CSS‐C1 at 0.1 A g^−1^ within voltage ranges of 0.01‐3 V. d) Rate performance CSS‐C0, CSS‐C1, and CSS‐C2, respectively. Cycling performance at e) 0.5 A g^−1^ and f) 10 A g^−1^ of CSS‐C0, CSS‐C1, and CSS‐C2, respectively. g) XRD patterns of CSS‐C1 anodes after different cycles.

Electrochemical evaluation within the voltage range of 0.01–3 V were performed to investigate how different synthesis process influence the properties of SIB anode materials. Cyclic Voltammetry (CV) curves (**Figure** **5**a) exhibit multiple pairs of redox peaks, indicating a characteristic multi‐step reaction process. The possible reactions are as follows.^[^ ^48^, ^49^, ^50^ ^]^

(1) $$\mathrm{SnS}+2Na^{+}+2e^{-}↔\mathrm{Sn}+Na_{2}S$$

(2) $$Co_{9}S_{8}+xNa^{+}+xe^{-}↔Na_{x}Co_{9}S_{8}↔\mathrm{Co}+Na_{2}S$$

(3) $$Na_{x}Co_{9}S_{8}+\left(16-x\right)Na^{+}+\left(16-x\right)e^{-}↔9\mathrm{Co}+8Na_{2}S$$

(4) $$\mathrm{Sn}+yNa^{+}+ye^{-}↔Na_{y}\mathrm{Sn}$$

During the first cathodic scan, the peak at 0.82 and 0.58 V are related to reactions of SnS and Co_9_S_8_, accompanied by the formation of a solid electrolyte interface (SEI) layer.^[^ ^51^, ^52^, ^53^, ^54^ ^]^ The region between 0 and 0.15 V can be attributed to the complex multi‐step alloying process between Sn and Na, while corresponding dealloying process is indicated by anodic peaks at 0.15 and 0.67 V. As shown in Figure 5b, the ex situ XRD patterns at specific voltages further confirm the above conclusion and also correspond to previous reports.^[^ ^55^, ^56^, ^57^ ^]^ In subsequent scans, the cathodic peaks at 1.2 and 0.9 V are attributed to the conversion reactions between sodium and SnS, as well as Co_9_S_8_, respectively.^[^ ^50^, ^53^ ^]^ Peaks below 0.7 V corresponds to multi‐step alloying reaction of Na with Sn. The range of ≈1.0–1.31 V is indication for the re‐formation of the SnS.^[^ ^38^ ^]^ Additionally, the peak at 1.62 V can be attributed to the reverse conversion reaction of Co_9_S_8_. A minor current increase near 1.9 V may be linked to reactions involving the carbon coating.^[^ ^58^ ^]^ The following CV curves show a high degree of overlap, indicating strong electrochemical reversibility during repeated insertion/extraction. Despite the similarity of the corresponding electrochemical properties of the other two samples (Figure S4, Supporting Information), the enhanced curve consistency of CSS‐C1 indicates its superior stability in sodium ion storage. Therefore, the one‐step process of CSS‐C1 enhances the stable transport and storage of sodium ions. The platforms of charge/discharge curves (Figure 5c) closely match the CV analysis. The consistent shape across cycles demonstrates that CSS‐C1 maintains excellent reversibility during cycling. Notably, the first discharge and charge capacities were 887.6 and 796.6 mAh g^−1^, respectively, indicating a small capacity loss. In contrast, the first discharge/charge capacities were 1084.6/981.5 mAh g^−1^ for CSS‐C0 and 870.2/755.4 mAh g^−1^ for CSS‐C2 (Figure S4, Supporting Information). Less capacity loss means higher active material utilization and stability as SIB anodes.

The rate performance of different anodes was evaluated at a large current density range from 0.1 to 20 A g⁻¹. As shown in Figure 5d, CSS‐C1 achieved impressive reversible charging capacities of 882.4, 785.6, 580.5, 490.9, 402.9, 358.8, 278.5, and 220.4 mAh g^−1^ at current densities of 0.5, 1, 3, 5, 8, 10, 15, and 20 A g^−1^, respectively. The comparative results clearly indicate that the carbon coating enhances stability, while the one‐step process further boosts stability at high current densities. Although all three exhibit close capacity at initial current densities, the specific capacity of CSS‐C1 remain consistently higher than those of CSS‐C0 and CSS‐C2 at all tested current densities. Notably, CSS‐C1 retains a high specific capacity of 220.4 mAh g^−1^ at a high current density of 20 A g^−1^, while CSS‐C2 remains to this capacity at 15 A g^−1^, and CSS‐C0 keeps to this level at 10 A g^−1^. This indicates that the one‐step synthesized CSS‐C1 significantly enhances capacity retention, particularly at high current densities. It also highlights that while carbon coating improves the active material's stability and rate performance, the enhancement varies with different carbonization process. Optimized synthesis process is crucial for maximizing both stability and rate performance. Moreover, even after cycling 5 cycles at the high current density of 20 A g^−1^, all samples rapidly recover to ≈760 mAh g^−1^ when the current density returns to 0.5 A g^−1^, indicating excellent reversible stability for sodium ion storage.

As shown in Figure 5e, the CSS‐C0 exhibited the most significant decay, failing after only 70 cycles. In contrast, both CSS‐C1 and CSS‐C2 sustained 100 charge/discharge cycles at 0.5 A g^−1^ without failure, albeit with reduced capacity. This highlights the effectiveness of carbon‐coating in enhancing stability and minimizing capacity loss.^[^ ^50^ ^]^ Their decay could be attributed to the activation and structural reconstruction of the active material, processes that typically require long observation periods at lower current densities. Hence, Figure 5f further demonstrates the cycling stability at a high current density of 10 A g^−1^. All three samples exhibit a decline followed by an increase in capacity before stabilizing. This behavior can be explained as the activation and restructuring of active materials. The areal capacity is calculated in Table S1 (Supporting Information). It also corresponds well with the XRD patterns in Figure 5g. The XRD peak intensity decreases initially and then increases as cycling progresses, indicating potential structural restructuring within the material. Notably, CSS‐C1 stabilizes after only 250 cycles, whereas CSS‐C0 takes 600 cycles, and CSS‐C2 requires 658 cycles to reach stabilization. This indicates that the one‐step treatment effectively promotes restructuring and accelerates the activation process. CSS‐C1 maintains a high capacity of 389 mAh g^−1^ (90.2% capacity retention) even after 2300 cycles, whereas CSS‐C2 fails after 1288 cycles, and CSS‐C0 only lasts for 600 cycles. To better demonstrate the advantages of our work, Table S2 (Supporting Information) shows a comparison between our performance and reported Co/Sn‐based SIB anodes. As shown in the table, our work achieves the highest combination performance of rate performance and stability. It maintains a high capacity of 220.4 mAh g^−1^ at an ultra‐high current density of 20 A g^−1^, significantly outperforming comparable materials. Also, it can be stabilized for 2300 cycles at a high current density of 10 A g^−1^. The enhanced stability can be attributed to the advantages in synthesis methods. As we mentioned before, one‐step synthesis process can retain the optimal morphology and enhance the C‐S bond, which result in electron transfer and rate performance. This comparison implies that CSS‐C1 can be used as advanced SIB anodes with high stability, and also illustrates that the optimized synthesis process can effectively improve the stability and rate performance of active material.

**Figure 6:**
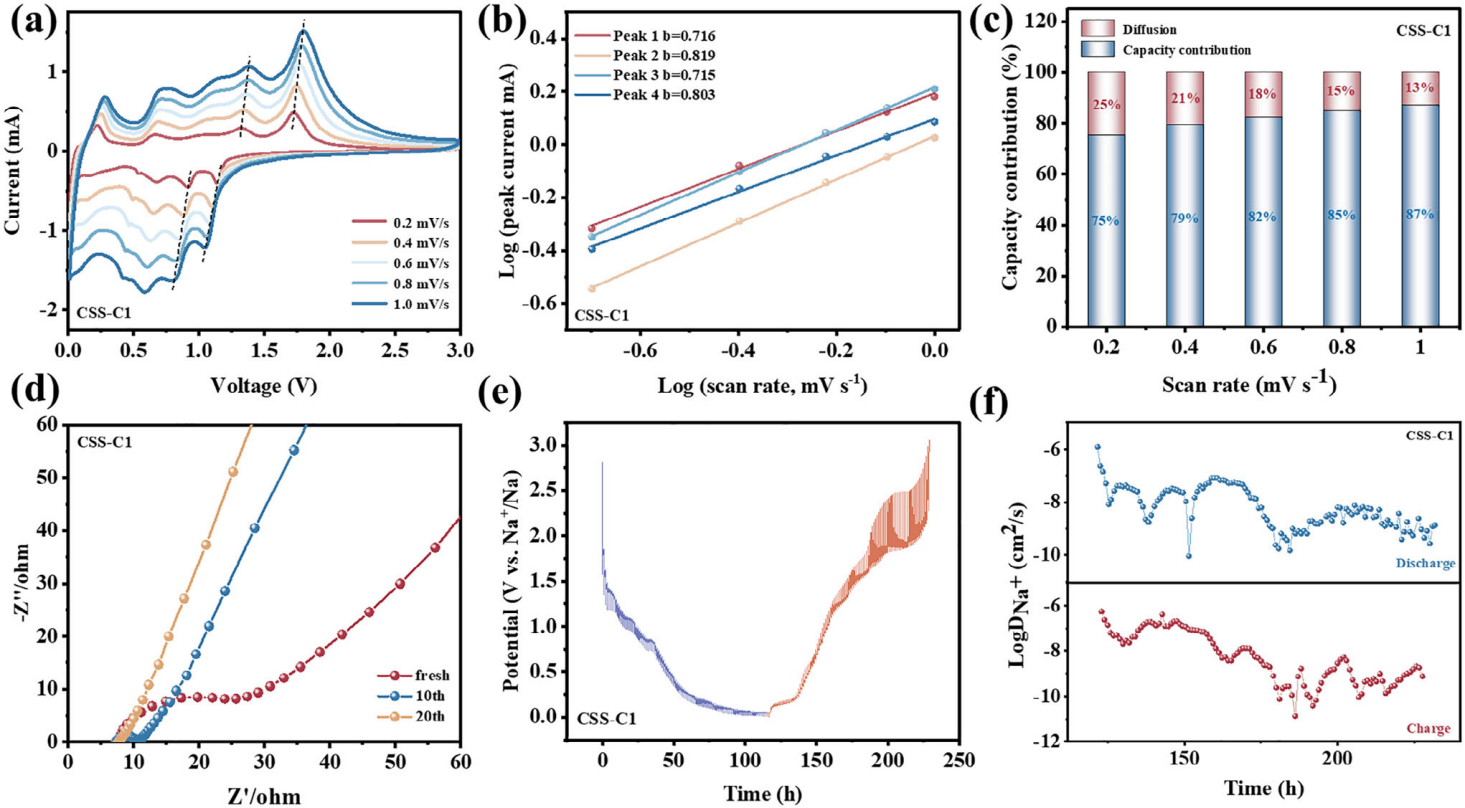
a) CV curves of CSS‐C1 at scanning rates from 0.2 to 1.0 mV s^−1^. Corresponding b) Log(*i_p_*) versus Log(*v*) plots, and c) capacitive contribution percentages, respectively. d) EIS curves of CSS‐C1 anodes after different cycles. e) GITT curve in discharged/charged state and f) calculated sodium ion diffusion coefficient for CSS‐C1, respectively.

To better understand the performance enhancement, CV at different scan rates were conducted to analyze the electrochemical kinetics of active materials as SIB anodes. The good stability can be indicated by a similar shape shown in all the CV curves from 0.2 to 1.0 mV s^−1^ (**Figure** **6**a). The charge storage mechanism is identified by the equation (*i = av^b^*). The value of *b* can be determined from the relationship between the peak currents (*i*) and the scan rates (*v*). A *b*‐value approaching 1 indicates that the capacitive contribution is dominant, while it closer to 0.5 suggests that diffusion‐controlled processes is dominant. As shown in Figure 6b, CSS‐C1 demonstrates a high *b*‐value, indicating a dominant capacitive contribution during sodium ion storage. This usually means a faster dynamic response, which supports improved rate performance and enhanced capacity retention. In contrast, CSS‐C2 has a lower b‐value, suggesting a higher diffusion‐controlled contribution to its storage mechanism compared to CSS‐C1. It indicates that its kinetics are constrained by sodium‐ion diffusion in the electrode material (Figure S5a,b, Supporting Information). Therefore, optimizing electrode material by enhancing capacitive dominated behavior, reducing diffusion resistance, and refining electrode structure may effectively improve the overall performance of sodium‐ion batteries. Figure 6c further analyzes the percentage of capacitive contribution of CSS‐C1. The capacitive contribution derived from the CV curves reaches 75% at a scan rate of 0.2 mV s^−^¹, increasing further to 87% as the scan rate rises. In comparison (Figure S5c, Supporting Information), the capacitive contribution of CSS‐C2 rises from 67% to 82%, highlighting the superior rate performance of CSS‐C1. Additionally, the electrochemical impedance spectroscopy (EIS) plots (Figure 6d; Figure S6, Supporting Information) after different cycles were employed to investigate electrochemical behavior of samples. The plots show a similar shape with a semicircle in the high‐frequency region, representing charge transfer resistance (R_ct_), and a line in the low‐frequency region, corresponding to Warburg impedance (W).^[^ ^37^ ^]^ Detailed data are shown in Table S3 (Supporting Information). While R_ct_ decreases with the number of cycles for all three samples, CSS‐C1 shows a more rapid reduction and maintains lower resistance overall, this indicates that electron and ion transport is more efficient, contributing to improved overall electrochemical performance. Notably, the charge transfer impedance reduction of CSS‐C1 may be due to the one‐step synthesis method that optimizes the interface between the carbon coating and the active material, improves the electronic conductivity, and promotes the interfacial charge transport process. In addition, after 20 cycles, the slope of CSS‐C1 in the low frequency region increased significantly, which further proved that its sodium ion diffusion rate was fast, which was conducive to enhancing the stability of long cycles. The kinetic behavior of sodium ion diffusion in CSS‐C1 was further examined using the Galvanostatic Intermittent Titration Technique (GITT) shown in Figure 6e. The calculated sodium ion diffusion coefficient for CSS‐C1 (Figure 6f) is ranged from 10^−13.5^ to 10^−8^ cm^2^ s^−1^, significantly exceeding those of CSS‐C0 and CSS‐C2 (Figure S7, Supporting Information). Hence, the one‐step treatment significantly improves charge transfer and ion diffusion in the CSS‐C1 anode, leading to outstanding rate performance and cycling stability.

**Figure 7:**
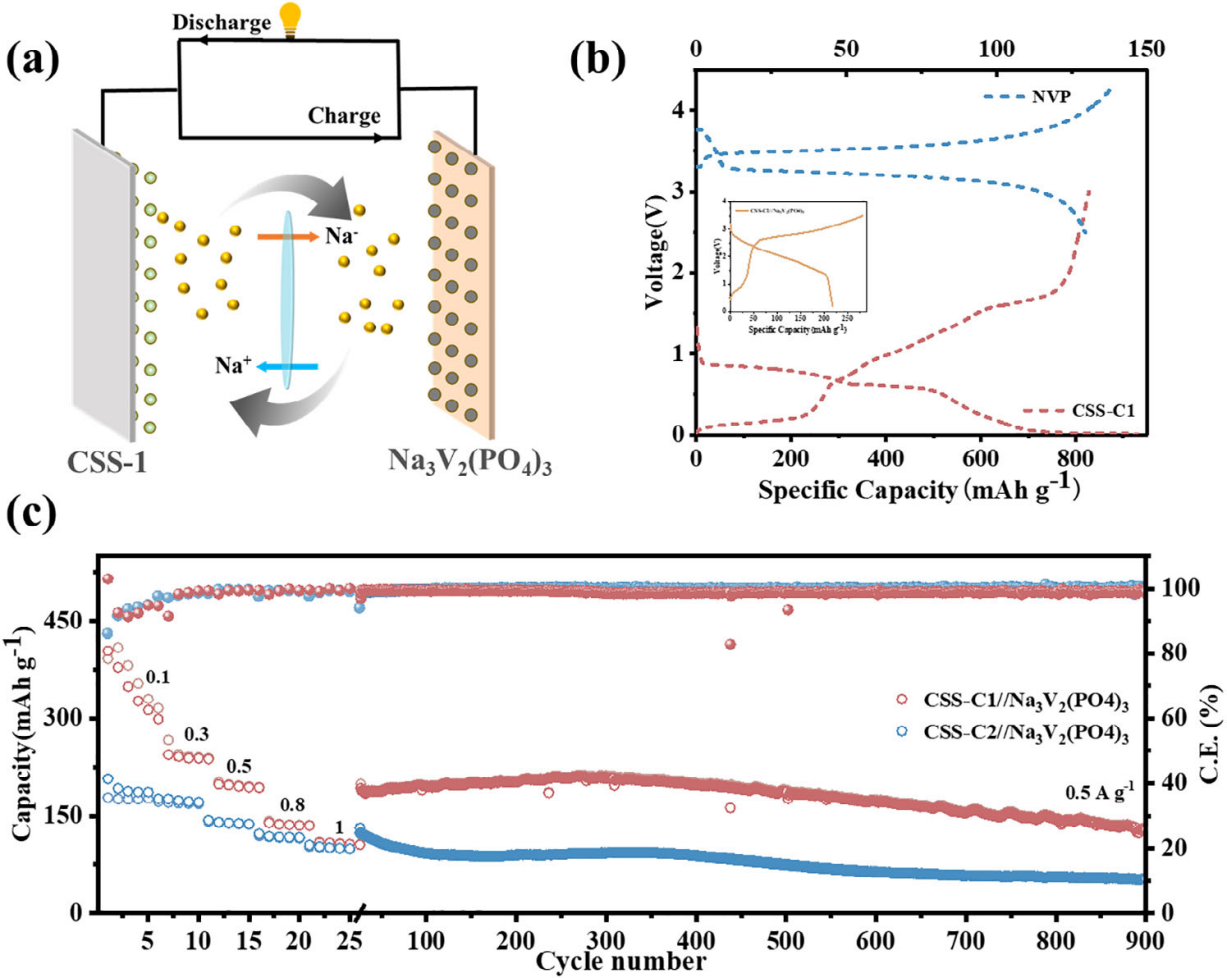
a) Schematic illustration of the CSS‐C1//Na_3_V_2_(PO_4_)_3_ full‐cell and the electrochemical performance: b) Charging/discharging curves of NVP and CSS‐1 t 500 mA g^−1^. The inset shows the charge/discharge curve of the full cell. c) Rate performance and Cycling performance during 900 cycles at 500 mA g^−1^.

As shown in **Figure** **7**a, the full cell (CSS‐C1||Na_3_V_2_(PO_4_)_3_) was assembled for further demonstration of its practicality. As shown in Figure 7b, with CSS‐C1 showing a discharge plateau ≈0.5 V and Na_3_V_2_(PO_4_)_3_ (NVP) exhibiting a charge plateau near 3.5 V, the full cells were investigated within an optimized voltage window of 0.2–3.5 V. The output voltage of the full cells of only ≈2 V Figure S9 (Supporting Information). Figure 7c shows the integrated rate performance and cycling stability of the full cell. It is realized by performing a continuous cyclic charge/discharge test directly after the rate performance test. The electrochemical behavior of the NVP is shown in Figure S9 (Supporting Information). The full cell demonstrated a capacity of 300, 238.5, 193.1, 134.9, and 106.5 mAh g^−1^ at current densities of 0.1, 0.3, 0.5, 0.8, and 1 A g^−1^, respectively. When the current density was returned to 0.5 A g^−1^, the capacity was restored to 188.5 mAh g^−1^, demonstrating the excellent stability of the full cell. At a current density of 0.5 A g^−1^, the cell consistently delivers a stable capacity of 130.5 mAh g^−1^ for up to 900 cycles. In contrast, the CSS‐C2 only retain a capacity of 53 mAh g^−1^. The full‐cell comparison further illustrates that rational optimization of the synthesis process can further maximize specific energy density and stability.

## 3 Conclusion

To explore the impact of synthesis process on the performance of bimetallic sulfide anodes, promising Co‐Sn sulfides were chosen for investigation, with samples prepared using different sequences of carbonation and sulfidation. The CSS‐C1 anode, synthesized via a one‐step simultaneous carbonation and sulfidation process, demonstrated enhanced sodium‐ion storage kinetics and improved electrical conductivity, resulting in superior stability and rate performance. This approach enabled the CSS‐C1 anode to retain a capacity of 220.4 mAh g^−1^ at an ultra‐high current density of 20 A g^−1^ and maintain 389 mAh g^−1^ after 2300 cycles at 10 A g^−1^. Furthermore, when paired with Na_3_V_2_(PO_4_)_3_ in a full‐cell configuration, the battery achieved stable cycling over 900 cycles with a capacity of 130.5 mAh g^−1^. Our research aims to explain the impact of the sulfidation‐carbonization process on performance, thereby advancing the development of stable and high‐performing SIB anodes.

## Conflict of Interest

The authors declare no conflict of interest.

## Supporting information



Supporting Information

## Data Availability

The data that support the findings of this study are available from the corresponding author upon reasonable request.
