# Supplementary material for: Enhanced Stability of Sodium‐Ion Batteries by Controlling the Synthesis Process of Binary Metal Sulfides
Source: Small. 2025 Mar 27;22(12):2412776. doi: 10.1002/smll.202412776 (PMC12934358; doi:10.1002/smll.202412776)
Supplement: Supplementary file 1 — Supporting Information [file SMLL-22-2412776-s001.docx]

Supporting Information

Enhanced stability of sodium-ion batteries by controlling the synthesis process of binary metal sulfides

Wenbo Qiu, Zidong Wang, Huaping Zhao, Yonglong Sheng, Guosheng Shao*, and Yong Lei*

Experimental Section

*Chemical reagents:* Tin chloride pentahydrate (SnCl_4_·5H_2_O, AR, 99.9%), cobalt chloride hexahydrate (CoCl_2_·6H_2_O, AR, 99.9%), Tris (hydroxymethyl) methyl aminomethane (THAM, AR), sodium hydroxide (NaOH, AR), dopamine hydrochloride (C_8_H_11_NO_2_·HCl, AR), citric acid, sodium salt (C_6_H_5_Na_3_O_7_·2H_2_O, AR), were purchased from Sinopharm Chemical Reagent.

*Synthesis of precursor:* 1 mmol SnCl_4_·5H_2_O was dissolved in 5 mL anhydrous ethanol, denoted as Solution A. Then 1 mmol CoCl_2_·6H_2_O and C_6_H_5_Na_3_O_7_·2H_2_O was dissolved in 35 mL deionized water, denoted as Solution B. Solution A was added to Solution B and mixed solution was stirred for 15 min. Then 5 mL NaOH with the concentration of 2 M was slowly dropped into mixed solution. After being stirred for 1 h, the product was washed and dried for 12 h. The final product was precursor CoSn(OH)_6_.

*Synthesis of CSS-C0:* At first, precursor CoSn(OH)_6_ was directly transferred with sulfur powder into a tube furnace with a mass ratio of 1:3. After heated it to 600°C with a rate of 5 ℃ min^-1^ for 2h under a mixed Ar_2_/H_2_ atmosphere, the final product was named as CSS-C0.

*Synthesis of CSS-C1:* 100 mg of the precursor was dispersed into 100 mL buffer solution with 10 mmol Tris. After being under ultrasound for 10 min, 70 mg dopamine hydrochloride was added to the mixture and stirred for 12 h. The samples were collected by centrifuge and drying. Finally, the sample and sulfur powder were placed in a tube furnace with a mass ratio of 1:3, then heated to 600°C at a rate of 5 ℃ min^-1^, under a mixed Ar_2_/H_2_ atmosphere for 2 h. The final product was named as CSS-C1.

*Synthesis of CSS-C2:* Firstly, CSS-C0 was dispersed into 100 mL buffer solution dissolved with 10 mmol Tris. After being under ultrasound for 10 min, 70 mg dopamine hydrochloride was added and then stirred for 12 h. The samples were collected by centrifuge and drying. Then it was placed into a tube furnace with sulfur powder. After heated to 600 ℃ with a rate of 5 ℃ min^-1^, it was kept for 2 h under Ar atmosphere. The final product was named as CSS-C2.

*Materials Characterization:* X-ray diffraction (XRD, 3KW D/MAX2200V PC, Rigaku, Japan) was used to confirm the crystal structure. The morphology was characterized by using scanning electron microscopy (SEM, Nova nano F7500, FEI, USA) and transmission electron microscopy (TEM, JEOL JEM-2100F). Energy dispersive spectroscopy (EDS, OXFORD Xplore, OXFORD Instruments) was used to measure the elemental distribution of the material at an accelerating voltage of 15 kV. X-ray photoelectron spectroscopy (XPS, Thermo Scientific K-Alpha, Waltham, MA, USA) was used to investigated the chemical elements.

**Electrochemical measurements:**

*Half-cell assessment and investigation:* The coin cells were assembled in an argon-filled glove box. At first, active materials, Super P and sodium carboxymethyl cellulose were mixed with the mass ratio of 7:2:1. The obtained mixed slurry is coated to the copper foil and dried under vacuum at 70 °C for 12 h. Sodium metal piece was served as the counter/reference electrode, and glass fiber was used as separator. The electrolyte was 1 M NaPF_6_ in ethylene glycol dimethyl ether (DME). Then the cells were tested on the Maccor Series 4000 battery testing system with different working voltages. The active material mass loading is about 1.0 mg. Cyclic voltammetry (CV) and electrochemical impedance spectroscopy (EIS) were investigated by a multi-channel electrochemical analyzer (VMP3). CV plots were measured at different scanning rates in the range of 0.01 to 3 V. EIS was investigated in the frequency range of 0.01 to 300 kHz. The sodium diffusion and conductivity properties were evaluated by GITT.

*Full cell assessment and investigation:* Coin-type full cells were assembled with CSS-C1/CSS-C2 anodes, electrolyte, separators, and Na_3_V_2_(PO_4_)_3_ cathodes. The potential range was selected as 0.2-3.5 V for full cell testing. The N/P ratio is about 1.2. The mass loading of active material is around 1.1 mg cm^-2^ for the anodes and around 5.26 mg cm^-2^ for the cathodes. The current densities are chosen from 0.1 to 1 A g^-1^. The specific capacity of full cells is obtained based on anode materials.

**Calculation details**

For ion storage mechanisms identification, ^[1]^

$$i=av^{b}$$

where, *i* is peak current and *v* is scanning rate. a and b are two constants. The closer the *b*-value is to 1, the more the capacitive control process dominates.

For quantitative differentiation of capacities for diffusion/capacitance controlled processes,

$$i\left( v \right)=k_{1}v+k_{2}v^{\frac{1}{2}}$$

where, *k_1_* and k_2_ are two indictors for different controlled processes. The formula can be deformed as follows.

$$\frac{i\left( v \right)}{v^{\frac{1}{2}}}=k_{1}v^{\frac{1}{2}}+k_{2}$$

For GITT analyses, ^[2]^

$$D=\frac{4}{\pi\tau}\left( \frac{m_{A}V_{m}}{M_{A}S} \right)^{2}\left( \frac{\Delta E_{s}}{\Delta E_{t}} \right)^{2}$$

where, *τ* is the relaxation time. *m_A_* is the mass loading and *V_m_* is the molar volume of the electrode material. *M_A_* is molar mass. *S* is the electrode/electrolyte contact area. *ΔE_s_* is the voltage change due to the pulse. *ΔE_t_* is the voltage change due to constant current charging (discharging).

[1] S. Xue, J. Shang, X. Pu, H. Cheng, L. Zhang, C. Wang, C.-S. Lee, Y. Tang, Energy Storage Materials 2023, 55, 33.

[2] N. Shi, Y. Chu, B. Xi, M. Huang, W. Chen, B. Duan, C. Zhang, J. Feng, S. Xiong, Advanced Energy Materials 2020, 10.


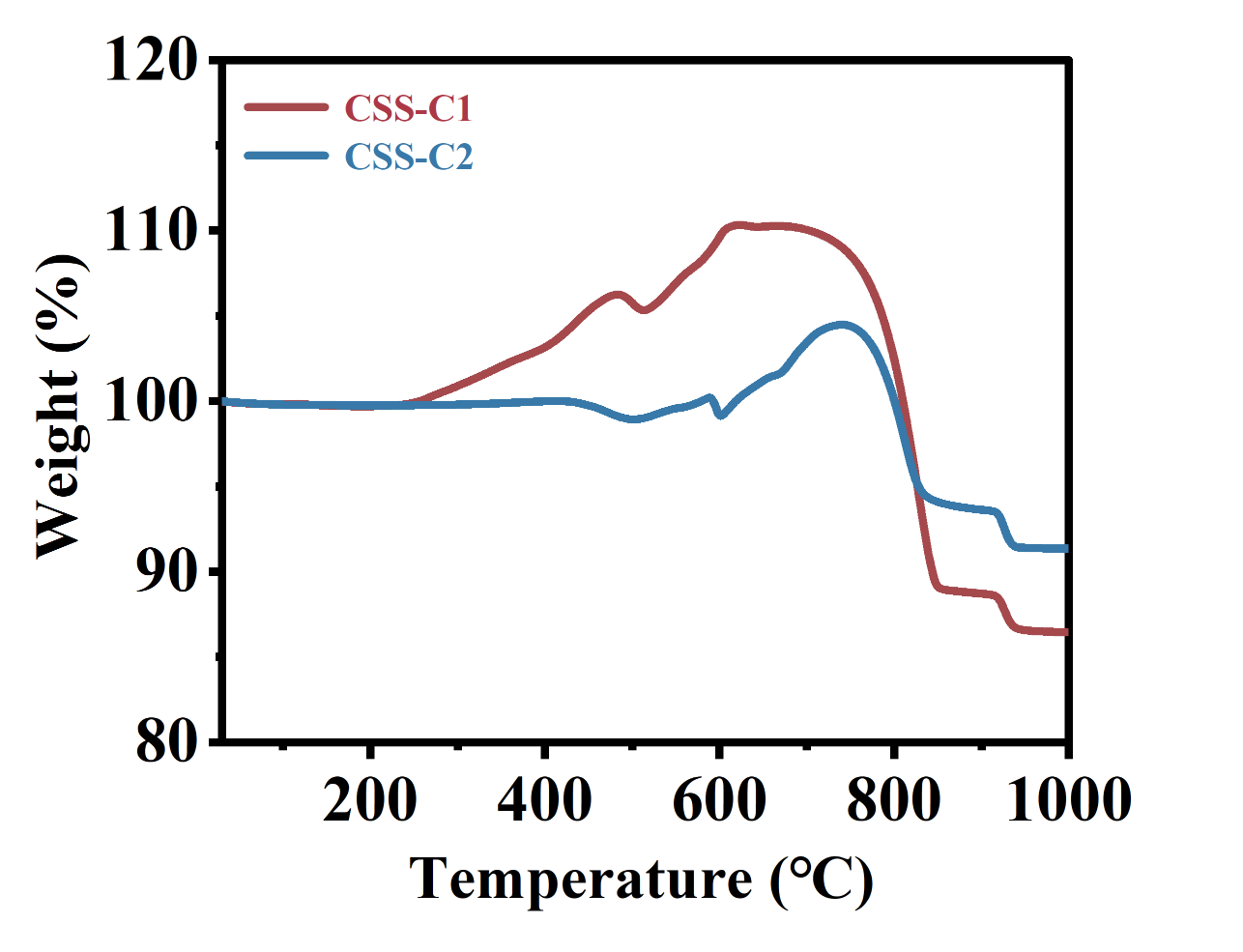


**Figure S1.** TG analysis of CSS-C1 and CSS-C2 composites at the temperature ranging from 20 to 800 ℃ in air.


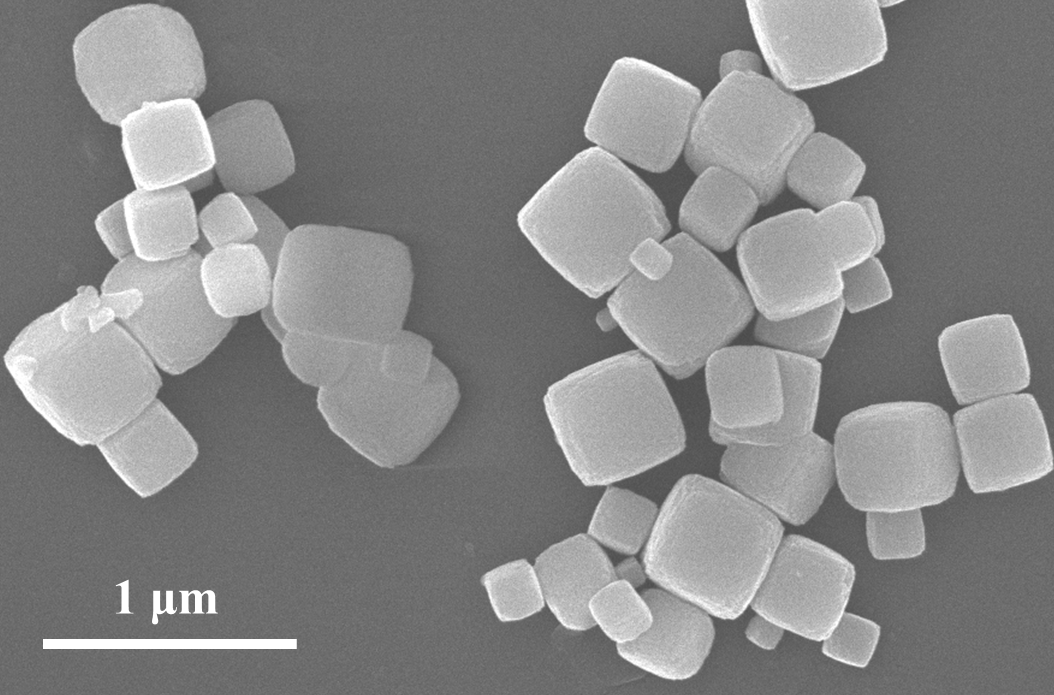


**Figure S2.** SEM image of CoSn(OH)_6_.


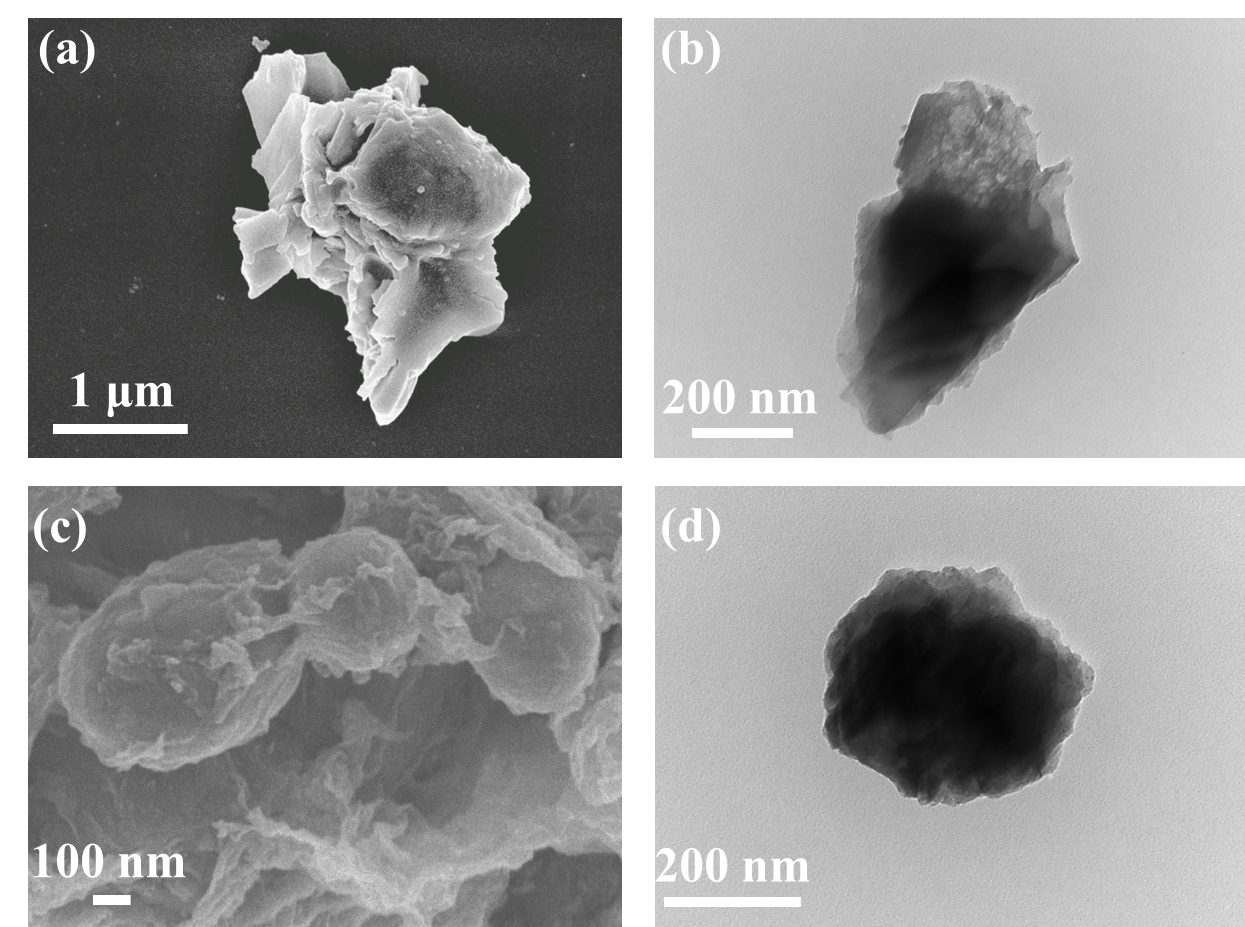


**Figure S3.** (a) SEM and (b) TEM images of CSS-C2. (c) SEM and (d) TEM images of CSS-C0.


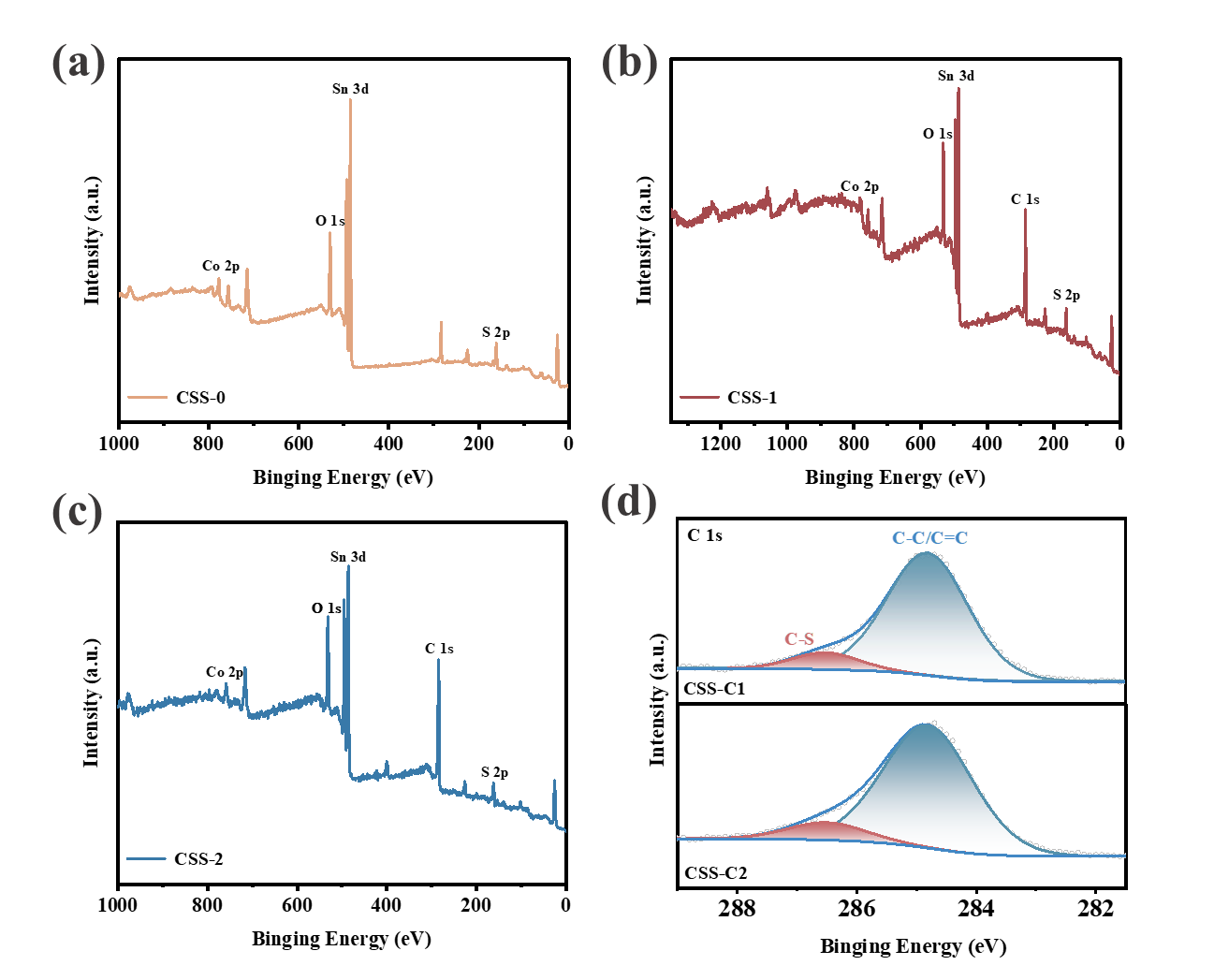


**Figure S4.** (a-c) Full XPS survey spectrum of CSS-0, CSS-1, and CSS-2, respectively. (d) C 1s spectrum of CSS-C1 and CSS-C2.


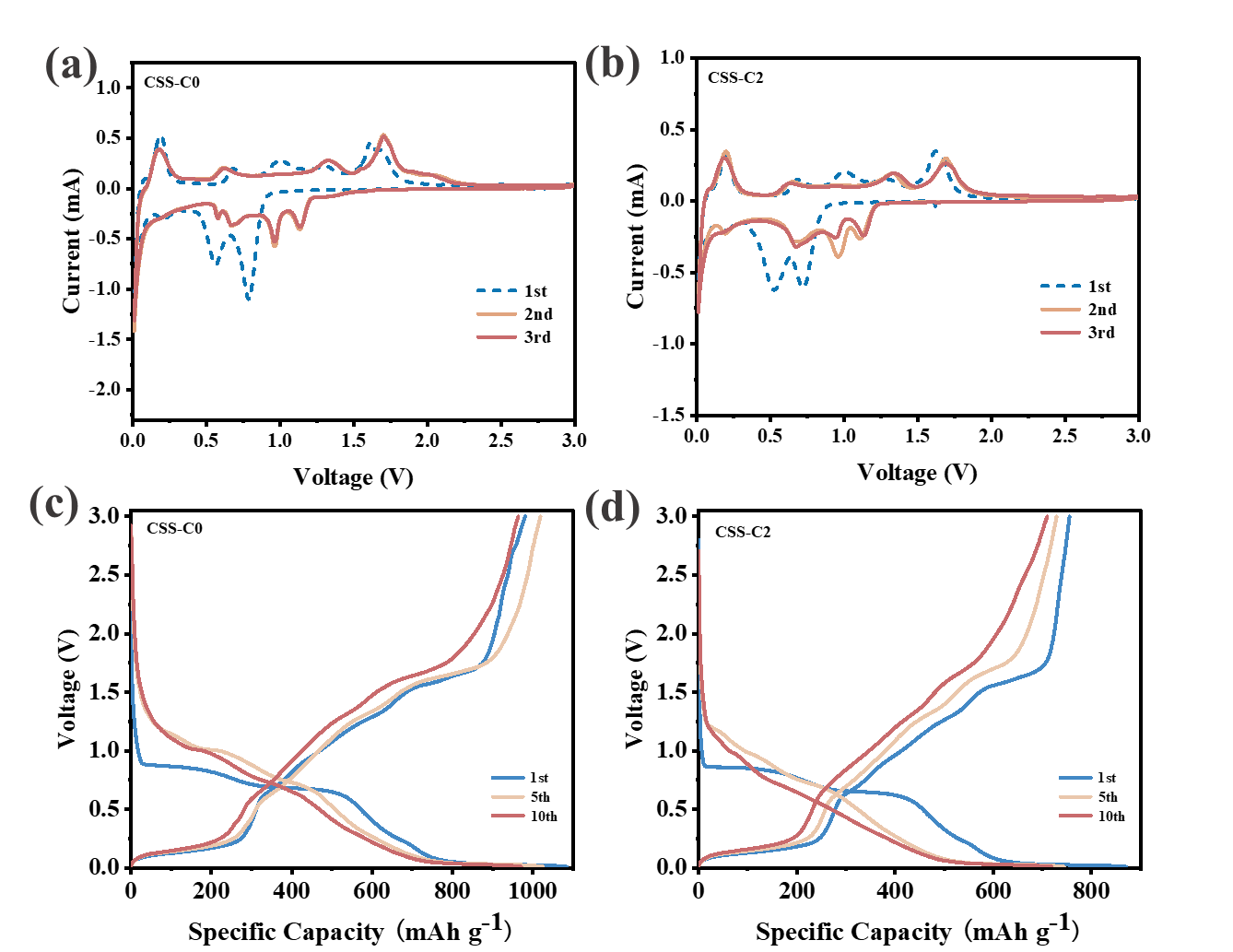


**Figure S5.** (a-b) CV curves and (c-d) discharge and charge curves of CSS-C0 and CSS-C2, respectively.


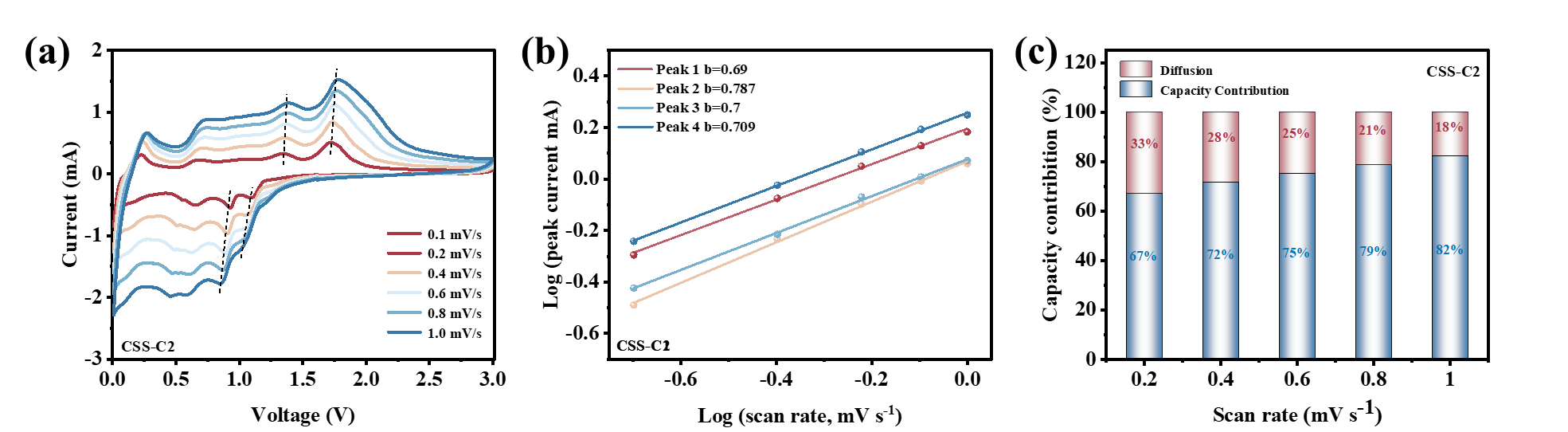


**Figure S6.** (a) CV curve at different scanning rates of CSS-C2, and corresponding (b) b-values. (c) Capacitive contribution of different CV curves of CSS-C2.


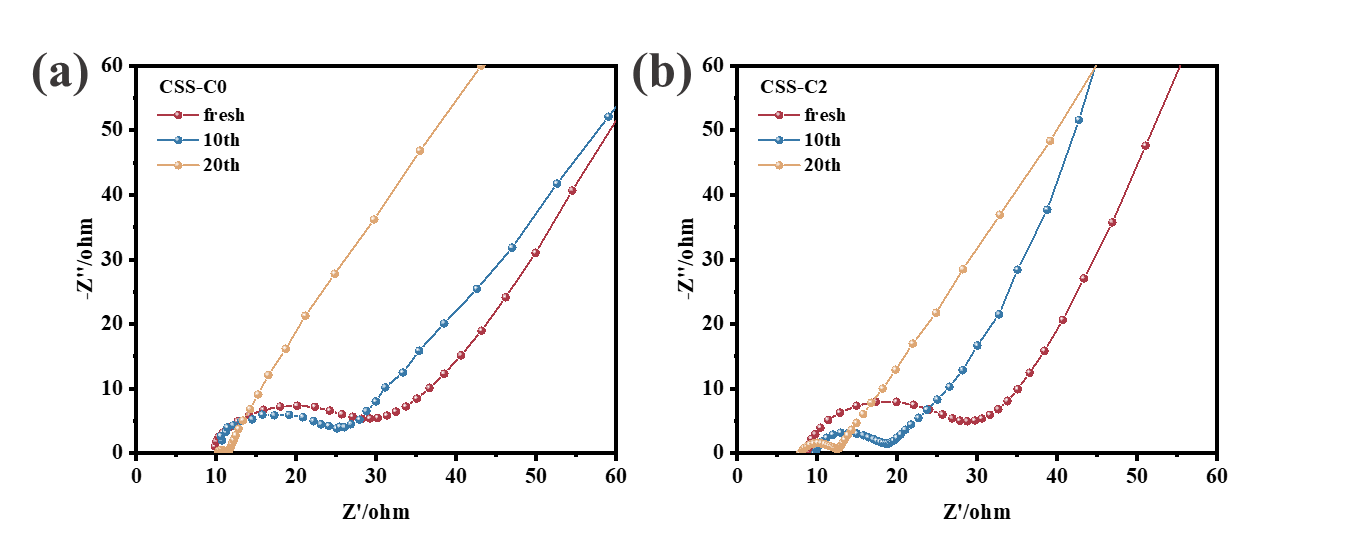


**Figure S7.** EIS plots of (a) CSS-C0 and (b) CSS-C2 after different cycles.


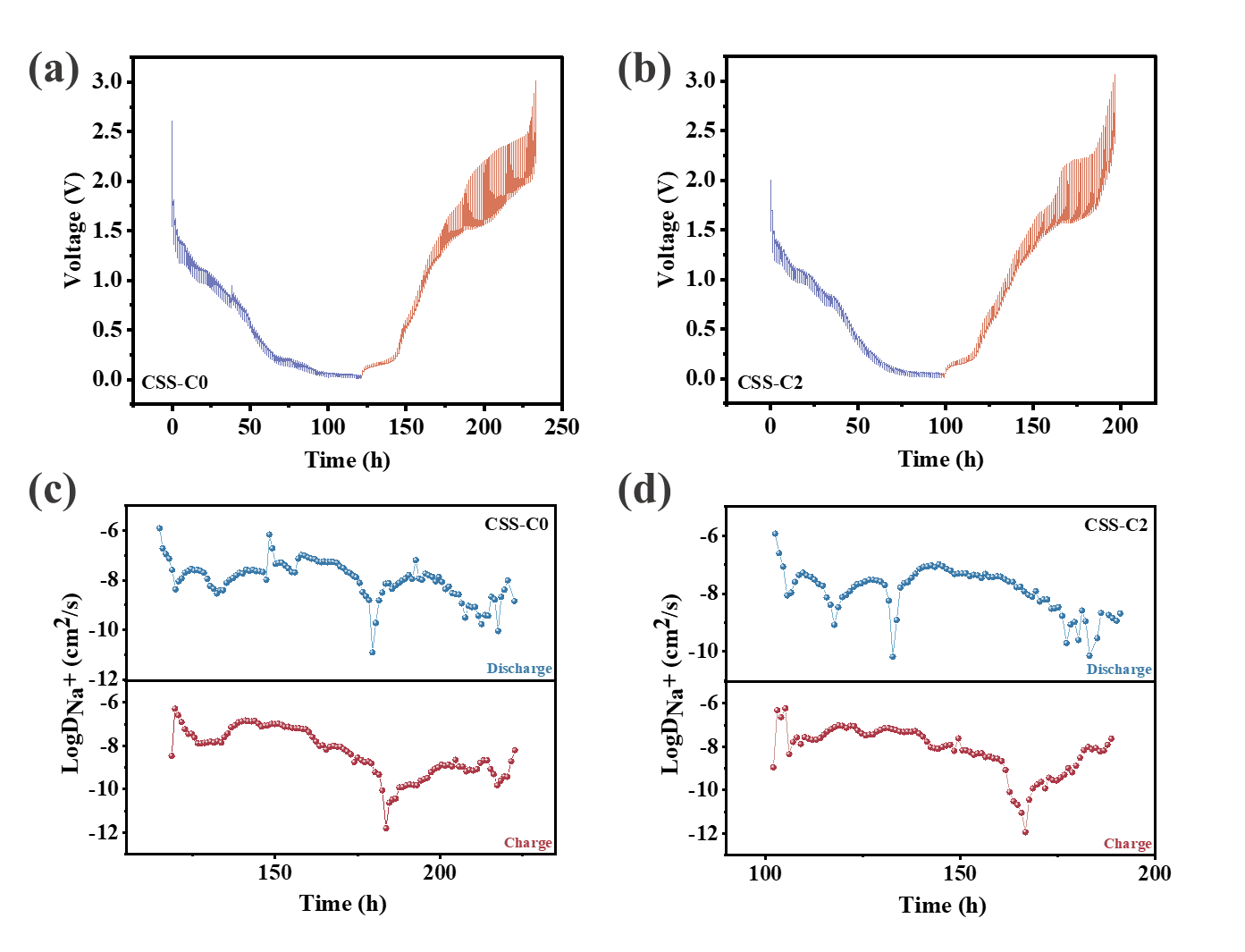


**Figure S8.** GITT plots of (a) CSS-C0 and (b) CSS-C2, respectively. The sodium-ion diffusion coefficients calculated from corresponding GITT plots of (c) CSS-C0 and (d) CSS-C2, respectively.

**Figure S9.** (a) The cycling performance of NVP at 0.5 A g^-1^. (b) The rate performance of NVP. (c) Charge/discharge curve of the full cell. **
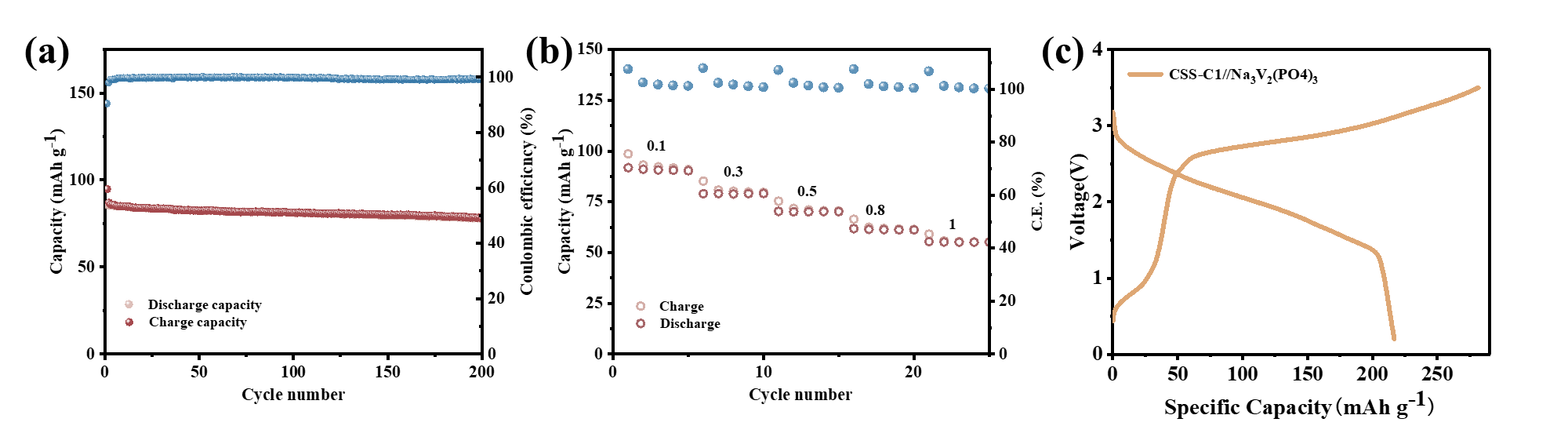
**

**Table S1.** Comparison of the sodium-ion storage performance between CSS and other Co/Sn-based anode materials.

|  | Current density (A g⁻¹) | Areal capacity (mAh cm^-2^) | Mass (mg) |
| --- | --- | --- | --- |
| CSS-C0 | 0.5 | 0.48 | 1.93 |
|  | 10 | 0.27 | 1.85 |
| CSS-C1 | 0.5 | 0.54 | 1.89 |
|  | 10 | 0.34 | 1.77 |
| CSS-C2 | 0.5 | 0.48 | 1.96 |
|  | 10 | 0.29 | 1.75 |

**Table S2.** Comparison of the sodium-ion storage performance between CSS and other Co/Sn-based anode materials.

| Anode materials | Voltage window  (V) | Rate capability | | Cycling stability | | | Ref |
| --- | --- | --- | --- | --- | --- | --- | --- |
|  |  | Capacity (mAh g^-1^) | Current density (A g^-1^) | Capacity (mAh g^-1^) | Current density  (A g^-1^) | Cycles  (number) |  |
| Co_9_S_8_/SnS | 0.005-3 | 354.5 | 2 | 339.2 | 0.5 | 1000 | ^[1]^ |
| SnS_2_@CoS_2_–rGO | 0.01-3 | 330 | 4 | 309.8 | 1 | 100 | ^[2]^ |
| SnS@Co_1-x_S | 0.01-3 | 352.92 | 10 | 546.56 | 1 | 60 | ^[3]^ |
| (SnCo)S_2_/SG | 0.2-3 | 502 | 5 | 487 | 5 | 5000 | ^[4]^ |
| CoS@SnS | 0.01-3 | 210 | 2 | 260 | 0.5 | 2000 | ^[5]^ |
| CoSnS_x_@NC | 0.01-3 | 170 | 5 | 180 | 1 | 4000 | ^[6]^ |
| (CoSn)S/C | 0.01-3 | 330 | 2 | 368 | 2 | 1000 | ^[7]^ |
| Sn–Co@void@C | 0.01-2 | 256 | 1 | 330 | 0.1 | 105 | ^[8]^ |
| r-Co_9_S_8_@NC | 0.4-2.8 | 432 | 10 | 317 | 5 | 1500 | ^[9]^ |
| Co_9_S_8_@C/NTs | 0.1-3 | 258 | 10 | 423 | 1 | 600 | ^[10]^ |
| HNBC@Co_9_S_8_@CNTs | 0.01-3 | 449.3 | 5 | 426.5 | 1 | 500 | ^[11]^ |
| Co_9_S_8_@C | 0.01-3 | 297 | 5 | 305 | 5 | 1000 | ^[12]^ |
| Co_9_S_8_@CYSNs | 0.2-2.5 | 100.5 | 10 | 100 | 10 | 800 | ^[13]^ |
| CN/SnS | 0.01-3 | 353 | 2 | 548.2 | 1 | 300 | ^[14]^ |
| 3D SnS/C | 0.01-3 | 220.9 | 5 | 427.4 | 0.1 | 100 | ^[15]^ |
| SnS@CNT | 0.01-3 | 492 | 5 | 660 | 5 | 500 | ^[16]^ |
| SnS QDs@NC | 0.01-3 | 172 | 4 | 172 | 1 | 500 | ^[17]^ |
| SnS_2_@C | 0.01-2.5 | 362 | 5 | 440 | 2 | 500 | ^[18]^ |
| **CSS-C1** | **0.01-3** | **220.4** | **20** | **389** | **10** | **2300** | **This work** |
| **CSS-C0** | **0.01-3** | **75.8** | **20** | **292.6** | **10** | **600** |  |
| **CSS-C2** | **0.01-3** | **151.3** | **20** | **326.6** | **10** | **1285** |  |

Reference

[1] J. Luo, S.-L. Xiang, D.-Y. Han, A. Liu, J. Cunha, G.-Y. Li, Z.-H. Hou, H. Yin, *Rare Met.* **2023**, *43*, 612.

[2] X. Wang, X. Li, Q. Li, H. Li, J. Xu, H. Wang, G. Zhao, L. Lu, X. Lin, H. Li, S. Li, *Nanomicro Lett.* **2018**, *10*, 46.

[3] Y. Zhang, Y. Jin, Y. Song, H. Wang, M. Jia, *Appl. Surf. Sci.* **2023**, *637*, 157938.

[4] C. Yang, X. Liang, X. Ou, Q. Zhang, H.S. Zheng, F. Zheng, J.H. Wang, K. Huang, M. Liu, *Adv. Funct. Mater.* **2019**, *29*, 1807971.

[5] W. Luo, Y. Feng, D. Shen, J. Zhou, C. Gao, B. Lu, *ACS Appl. Mater. Interfaces* **2022**, *14*, 16379.

[6] X. Liu, Y. Wang, Z. Wang, T. Zhou, M. Yu, L. Xiu, J. Qiu, *J. Mater. Chem. A.* **2017**, *5*, 10398.

[7] S. Wan, M. Cheng, H. Chen, H. Zhu, Q. Liu, *J. Colloid Interface Sci.* **2022**, *609*, 403.

[8] S. Liu, X.-Z. Li, B. Huang, J.-W. Yang, Q.-Q. Chen, Y.-W. Li, S.-H. Xiao, *Rare Met.* **2021**, *40*, 2392.

[9] X. Lin, X. Cui, H. Yan, J. Lei, P. Xu, J. Fan, R. Yuan, M. Zheng, Q. Dong, *ACS Sustain. Chem. Eng.* **2019**, *7*, 15729.

[10] X. Ma, X. He, L. Yu, N. Ahmad, Z. Tao, Z.X. Jiang, J.C. Liang, S. Zeng, L. Shi, G. Zhang, *Batteries & Supercaps* **2024**, 7.

[11] G. Yang, Z. Zhou, X. Liu, Y. Zhang, S. Wang, W. Yan, S. Ding, *Electrochim. Acta* **2023**, *441*, 141804.

[12] Y. Zhang, N. Wang, P. Xue, Y. Liu, B. Tang, Z. Bai, S. Dou, *Chem. Eng. J.* **2018**, *343*, 512.

[13] Y. Zhao, Q. Fu, D. Wang, Q. Pang, Y. Gao, A. Missiul, R. Nemausat, A. Sarapulova, H. Ehrenberg, Y. Wei, G. Chen, *Energy Storage Mater.* **2019**, *18*, 51.

[14] H. Yin, L. Jia, H.Y. Li, A. Liu, G. Li, Y. Zhu, J. Huang, M. Cao, Z. Hou, *J. Energy Storage* **2023**, *65*, 107354.

[15] P. Xue, N. Wang, Y. Wang, Y. Zhang, Y. Liu, B. Tang, Z. Bai, S. Dou, *Carbon* **2018**, *134*, 222.

[16] T.H.T. Luu, D.L. Duong, T.H. Lee, D.T. Pham, R. Sahoo, G. Han, Y.-M. Kim, Y.H. Lee, *J. Mater. Chem. A.* **2020**, *8*, 7861.

[17] G.K. Veerasubramani, M.-S. Park, J.-Y. Choi, D.-W. Kim, *ACS Appl. Mater. Interfaces* **2020**, *12*, 7114.

[18] Q. Sun, D. Li, L. Dai, Z. Liang, L. Ci, *Small* **2020**, *16*, 2005023.

**Table S3.** Comparison of kinetic parameters of different samples.

| Voltage range | | CSS-C0 | CSS-C1 | CSS-C2 |
| --- | --- | --- | --- | --- |
| *b*-value | Peak 1 | - | 0.716 | 0.69 |
|  | Peak 2 | - | 0.819 | 0.787 |
|  | Peak 3 | - | 0.715 | 0.7 |
|  | Peak 4 | - | 0.803 | 0.709 |
| R_ct_ (Ω) | fresh | 1.368 | 1.348 | 4.397 |
|  | 10^th^ | 15.28 | 6.454 | 15.87 |
|  | 20^th^ | 14.95 | 2.439 | 6.549 |
| $\text{D}_{\text{Na}^{\text{+}}}$  _(_cm^2^ s^-1^_)_ | Discharge | 10^-10.92^ - 10^-5.9^ | 10^-10.3^ - 10^-5.92^ | 10^-10.19^ - 10^-5.93^ |
|  | Charge | 10^-11.8^ - 10^-6.28^ | 10^-10.87^ - 10^-6.28^ | 10^-11.93^ - 10^-6.23^ |
